# miRNAs in the Box: Potential Diagnostic Role for Extracellular Vesicle-Packaged miRNA-27a and miRNA-128 in Breast Cancer

**DOI:** 10.3390/ijms242115695

**Published:** 2023-10-28

**Authors:** Cinzia Giordano, Felice Maria Accattatis, Luca Gelsomino, Piercarlo Del Console, Balázs Győrffy, Mario Giuliano, Bianca Maria Veneziani, Grazia Arpino, Carmine De Angelis, Pietro De Placido, Erica Pietroluongo, Francesco Zinno, Daniela Bonofiglio, Sebastiano Andò, Ines Barone, Stefania Catalano

**Affiliations:** 1 Department of Pharmacy, Health and Nutritional Sciences, University of Calabria, Via P. Bucci, Arcavacata di Rende (CS), 87036 Cosenza, Italy; felice.accattatis@gmail.com (F.M.A.); luca.gelsomino@unical.it (L.G.); piercarlo.delconsole@unical.it (P.D.C.); daniela.bonofiglio@unical.it (D.B.); sebastiano.ando@unical.it (S.A.); ines.barone@unical.it (I.B.); 2 Centro Sanitario, University of Calabria, Via P. Bucci, Arcavacata di Rende (CS), 87036 Cosenza, Italy; 3 Clinical Laboratory Unit, A.O. “Annunziata”, 87100 Cosenza, Italy; 4 Departments of Bioinformatics and Pediatrics, Semmelweis University, 1094 Budapest, Hungary; gyorffy.balazs@med.semmelweis-univ.hu; 5 TTK Cancer Biomarker Research Group, 1117 Budapest, Hungary; 6 Department of Clinical Medicine and Surgery, University of Naples Federico II, 80133 Naples, Italy; m.giuliano@unina.it (M.G.); grazia.arpino@unina.it (G.A.); carmine.deangelis1@unina.it (C.D.A.); piertro.deplacido@unina.it (P.D.P.); erica.pietroluongo@gmail.com (E.P.); 7 Department of Molecular Medicine and Medical Biotechnology, University of Naples Federico II, 80133 Naples, Italy; biancamaria.veneziani@unina.it; 8 Immunohaematology and Transfusion Medicine, A.O. “Annunziata”, 87100 Cosenza, Italy; f.zinno@aocs.it

**Keywords:** breast cancer, extracellular vesicles, miRNAs, miRNA-27a, miRNA-128

## Abstract

Circulating extracellular vesicle (EV)-derived microRNAs (miRNAs) are now considered the next generation of cancer “theranostic” tools, with strong clinical relevance. Although their potential in breast cancer diagnosis has been widely reported, further studies are still required to address this challenging issue. The present study examined the expression profiles of EV-packaged miRNAs to identify novel miRNA signatures in breast cancer and verified their diagnostic accuracy. Circulating EVs were isolated from healthy controls and breast cancer patients and characterized following the MISEV 2018 guidelines. RNA-sequencing and real-time PCR showed that miRNA-27a and miRNA-128 were significantly down-regulated in patient-derived EVs compared to controls in screening and validation cohorts. Bioinformatics analyses of miRNA-target genes indicated several enriched biological processes/pathways related to breast cancer. Receiver operating characteristic (ROC) curves highlighted the ability of these EV-miRNAs to distinguish breast cancer patients from non-cancer controls. According to other reports, the levels of EV-miRNA-27a and EV-miRNA-128 are not associated with their circulating ones. Finally, evidence from the studies included in our systematic review underscores how the expression of these miRNAs in biofluids is still underinvestigated. Our findings unraveled the role of serum EV-derived miRNA-27a and miRNA-128 in breast cancer, encouraging further investigation of these two miRNAs within EVs towards improved breast cancer detection.

## 1. Introduction

Breast cancer represents the most commonly diagnosed malignancy, and the leading cause of cancer-related deaths among women worldwide, reaching around 684,996 deaths in 2020 [1]. Being a highly heterogeneous disease at both the morphological and molecular levels, breast cancer is associated with different clinical behaviors and treatment responses. Currently, this neoplasia is classified into distinct subtypes based on tumor staging/grading and the expression of the estrogen receptor (ESR1), progesterone receptor (PgR), human epidermal growth factor receptor 2 (HER2), and the proliferation marker Ki-67 [2]. Individual biomarker profiles may significantly influence tumor recurrence, drug resistance, and mortality, thus requiring different therapeutic approaches. Owing to this complexity, the identification of additional tools for the better management of patients with breast cancer remains a major challenge. 

Liquid biopsy, which analyzes biomarkers that tumors shed via body fluids, represents an actively emerging field in translational cancer research that enables dynamic patient monitoring and a more personalized medicine approach [3]. In this context, there is an exponential increase in interest in extracellular vesicles (EVs), which are small membrane-encapsulated particles released from a wide variety of cell types and recovered in most biological body fluids. EVs are important regulators of intercellular signaling in several physiological and pathological conditions, such as cancer, because they contain different types of bioactive molecules selectively derived from the cell of origin [4], including microRNAs (miRNAs) [5,6,7]. miRNAs, which are non-coding RNAs involved in several biological processes through their interaction with cellular messenger RNAs [8], play a key role in tumor development and progression by promoting the acquisition of cancer hallmark traits [9,10,11]. Compared to free circulating miRNAs, those packaged within EVs are well protected by the EV lipid bilayer membrane, thus improving their half-life and stability [12]. Therefore, EV-derived miRNAs have become particularly appealing, due to their stability and easy quantification in biological fluids, for diagnosis, prognosis, and therapeutic utility in oncological diseases [13,14]. Several studies have reported deregulated profiles of circulating EV-miRNAs in the serum of patients with breast cancer compared to healthy individuals. A correlation between EV-miRNA levels and the clinical classification, prognosis and patient’s outcome has also been described [14], further shifting the research focus from total-EV secretome studies, such as proteins, lipids, and nucleic acids, towards more specific molecular cargos within EVs, such as miRNAs. For instance, miRNA-1246 and miRNA-21 were detected at significantly higher levels in the plasma EVs of sixteen breast cancer patients compared to those of healthy subjects, and receiver operating characteristic (ROC) curve analysis indicated that their combination is a better indicator of breast cancer than each individual level alone [15]. Four miRNAs (miRNA-9, miRNA-16, miRNA-21, and miRNA-429) were found to be up-regulated in the EVs of early-stage breast cancer patients in comparison with those of healthy donors [16]. Another report showed that EV-derived miRNA-105 levels were elevated in metastatic breast cancer cases compared with non-metastatic cases and healthy donors, and elevated EV-miRNA-222 levels were observed in luminal A versus luminal B and triple-negative (TNBC) tumor subtypes [17], while reduced levels of serum EV-miRNA-17-5p were detected in eighty-three patients diagnosed with breast cancer compared to healthy women [18]. 11 EV miRNAs (i.e., miRNA-338-3p, miRNA-340-5p, miRNA-124-3p, miRNA-29b-3p, miRNA-20b-eta5p, miRNA17-5p, miRNA-130a-3p, miRNA-18a-5p, miRNA-195-5p, miRNA-486-5p, and miRNA-93-5p) were also associated with breast cancer recurrence without mirroring expression in primary tumor tissues [19]. In patients with TNBC undergoing neoadjuvant chemotherapy, a combined signature of four EV-miRNAs (miRNA-4448, miRNA-2392, miRNA-2467-3p and miRNA-4800-3p), identified by microarray analysis, was able to discriminate between patients with pathological complete response (pCR) and non-pCR [20]. More recently, a four-miRNA signature, before (miRNA-30b-5p, miRNA-328-3p, miRNA-423-5p, miRNA-127-3p) and after the first cycle of neoadjuvant chemotherapy (miRNA-141-3p, miRNA-34a-5p, miRNA-183-5p, miRNA-182-5p), has been found to correlate with the therapeutic outcome of breast cancer patients [21]. However, despite the increasing body of evidence on the potential of tumor-derived EV-miRNA biomarkers [22,23], their practical application is still unclear, mainly due to the highly variability of current methods for their accurate purification, isolation, and detection. Most studies in the literature target specific miRNAs by using RT-qPCR, or microarray platforms, which are restricted to the detection of miRNA molecules provided from genomic databases. In contrast, although more expensive and time-consuming, next-generation sequencing (NGS) technologies enable the measurement of the relative expression levels of miRNAs (over a wider dynamic range than is possible with microarrays), generating the unique possibility of identifying novel and not commonly studied miRNAs, and overcoming the limitations of the array-based approaches [24,25]. Moreover, its high sensitivity allows the profiling of low-input samples such as liquid biopsies, whose applications in diagnostics and prognostics have considerably expanded [26,27]. Here, we conducted NGS followed by qRT-PCR validation to examine the differences in the circulating EV-miRNA profiles of patients with breast cancer and those of healthy controls. The results of this study led to the identification of two significantly down-regulated EV-derived miRNAs, namely miRNA-27a and miRNA-128, in breast cancer with a potential diagnostic value. These findings were compared with the evidence from the studies included in a comprehensive systematic literature search.

## 2. Results

### 2.1. Isolation and Characterization of Extracellular Vesicles (EVs) Derived from Healthy Controls and Breast Cancer Patients

EVs were isolated from the blood serum of healthy subjects (C), and patients with breast cancer (BC), and their clinicopathological characteristics are summarized in Table 1.

**Figure 1:**
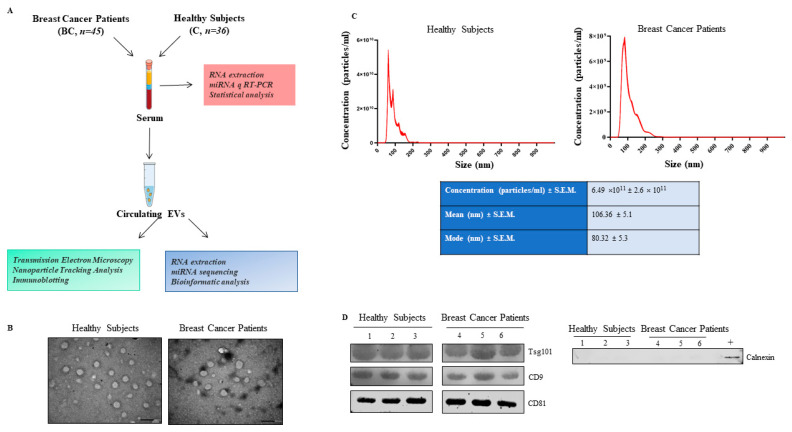
Characterization of circulating extracellular vesicles (EVs) isolated from sera of healthy subjects and breast cancer patients. (**A**). Schematic illustration of the study design. Circulating EVs were purified from serum samples of healthy subjects (C) and breast cancer patients (BC) by ExoQuick precipitation system. (**B**). Representative transmission electron microscopy (TEM) images of EVs isolated from serum of healthy subjects and breast cancer patients. Scale bar, 200 nm. (**C**). Upper panel, Representative size distribution profiles and concentration of serum EVs measured by nanoparticle tracking analysis (NTA). Lower panel, the table shows the average values for particle concentration and size from NTA results of the entire sample of specimens. (**D**). Immunoblot analysis showing expression of the EV canonical markers Tsg101, CD9, CD81 in equal amounts of EV lysates. The specificity of EVs isolation was confirmed using the endoplasmic reticulum marker Calnexin. +, MCF-7 breast cancer cell lysates were used as positive control.

Figure 1A shows a representative flowchart of the study. Circulating EVs were successfully obtained from the serum of our cohorts and characterized based on particle diameter, morphology, size distribution, and EV-marker enrichment according to the minimal experimental requirements for EVs [28]. TEM and NTA revealed the presence of oval or bowl-shaped EVs with a size range of 50–200 nm in the isolated fractions (Figure 1B,C), respectively. Immunoblot analysis showed that the EVs were enriched in canonical EV markers, including Tsg101, CD9 and CD81, whereas they were absent in the EV-negative control, such as the ER-associated protein calnexin (Figure 1D).

### 2.2. Transcriptomic Analysis of miRNAs Derived from EVs Isolated from Healthy Controls and Breast Cancer Patients

**Figure 2:**
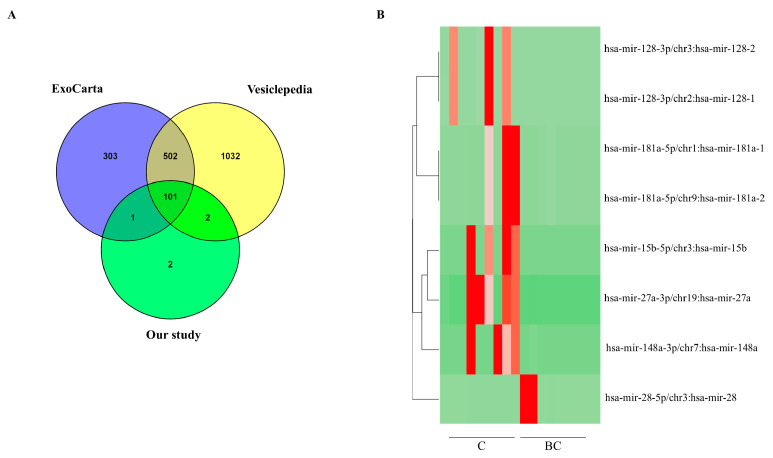
Differentially expressed EV-miRNAs in healthy subjects and breast cancer patients measured by RNA-seq. (**A**). Venn diagram of the known miRNAs in our study, vesicular Vesiclepedia and ExoCarta databases. (**B**). Heatmap of the differentially expressed EV-miRNAs in healthy subjects (C) and breast cancer patients (BC). The red and the green color scale indicate an increase and a decrease in miRNA expression levels, respectively.

Next-generation sequencing was then performed on the samples of our screening cohorts to define the global expression profile of the EV-miRNAs in the cases and controls. The EV samples of each group (n = 27) were mixed in equal amounts (3 samples in each pool) to obtain pooled EVs for breast cancer patients (n = 9 pools), and healthy women (n = 9 pools). It is well known that the number of miRNAs recovered into EVs is highly variable and low, since the conventional methods of EV-miRNA analysis and quantification can be plagued by inefficiencies [29]. In the present study, we identified a total of 135 miRNAs, and most miRNAs detected in our transcriptomic analysis were found in the vesicular databases of Vesiclepedia [30] and ExoCarta [31] (Figure 2A). Subsequent statistical assessment of the miRNAs identified by sequencing revealed that six miRNAs were significantly differentially expressed between healthy controls and patients with breast cancer. Specifically, the following miRNA was up-regulated: miRNA-28-5p (Fold Change = 102.18, padj = 2.59 × 10^7^), and the following five miRNAs were down-regulated: miRNA-27a-3p (Fold Change = −114.83, padj = 4.60 × 10^7^), miRNA-128-3p (Fold Change = −112.14, padj = 2.30 × 10^5^), miRNA-15b-5p (Fold Change = −1.88, padj = 1.00 × 10^4^), miRNA-181a-5p (Fold Change = −1.90, padj = 1.28 × 10^3^), and miRNA-148a-3p (Fold Change = −1.74, padj = 1.29 × 10^3^) in patients with breast cancer compared to the healthy population (Table 2). Figure 2B shows a heatmap of the results of the supervised hierarchical clustering. Based on the fold changes, miRNA-28-5p, miRNA-27a-3p, and miRNA-128-3p were selected for further validation by real-time PCR.

### 2.3. Comparison of the EV-Derived miRNA Signatures in the Validation Cohort and Bioinformatic Analysis for Selected miRNAs and Their Targets

To confirm the EV-derived miRNA signature identified in the screening cohort, we extended our analysis to a second series of healthy controls and patients with breast cancer (the validation cohort). The clinicopathological characteristics of the participants are presented in Table 3.

**Figure 3:**
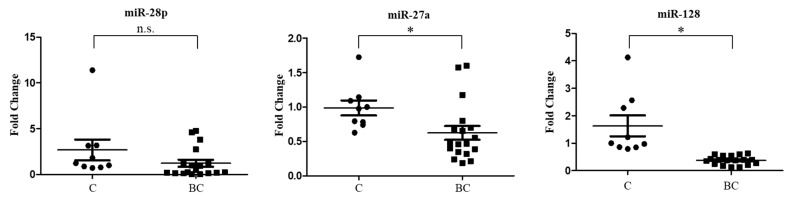
Quantitative real-time PCR (qRT-PCR) of miRNA expression profiling data in the validation cohort of healthy subjects (C) and breast cancer patients (BC). qRT-PCR was conducted to measure the miRNA expression levels of miRNA-28p, miRNA-27a and miRNA-128, as described in Materials and Methods. Data were reported as mean ± S.E.M. n.s. = not significant; * *p* < 0.05.

Therefore, EVs were isolated from the serum of these cohorts, and characterized using TEM, NTA and immunoblotting analyses, as described in the previous section. Real-time PCR assays confirmed a significant reduction in the levels of miRNA-27a-3p, and miRNA-128-3p in EVs of patients with breast cancer compared to healthy individuals. Conversely, no significant changes in the expression levels of miRNA-28-5p were observed in the validation cohort (Figure 3).

**Figure 4:**
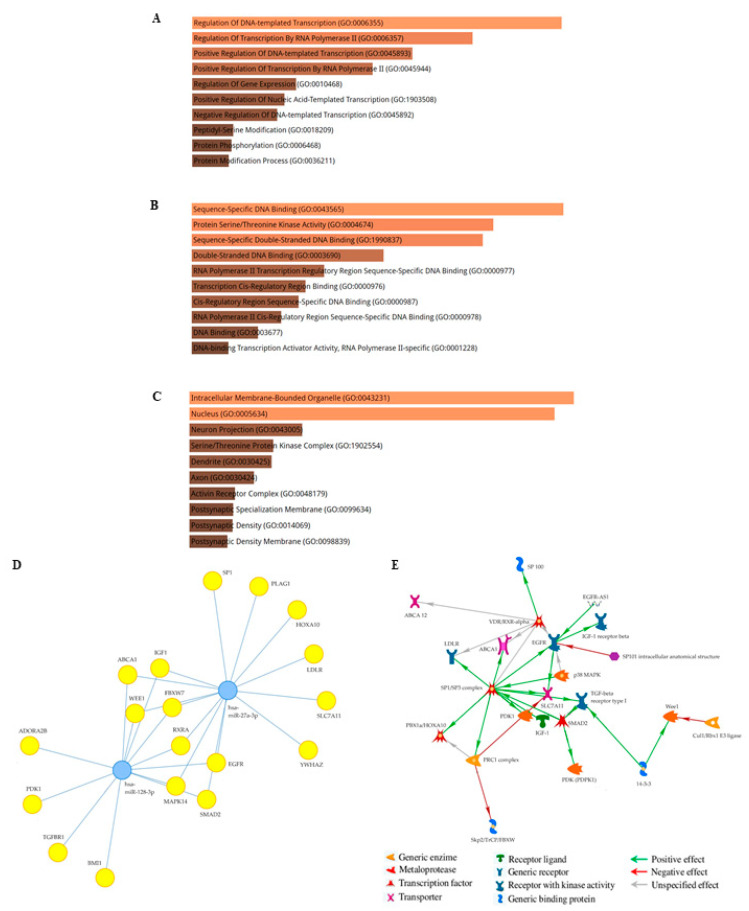
Bioinformatics analysis of the differentially expressed EV-miRNA target genes and miRNA-target interaction network for miRNA-27a and miRNA-128. Top 10 significant Enrichr GO terms for the (**A**) biological processes (BPs), (**B**) molecular functions (MFs) and (**C**) cellular component (CCs) of the 1781 target genes obtained by the miRDB target prediction database. The brightness and the length of the bars represent the significance of the category/term in terms of *p*-value. (**D**). The network shows the miRNA-target interactions retrieved from MIENTURNET for miRNA-27a and miRNA-128. Blue dots represent miRNAs, yellow dots represent miRNA targets. (**E**). MetaCore protein network built by processing 18 miRNA targets obtained by mirTarBase database. Functions of network objects are visualized by different symbols, as reported in the legend. Edge colors and arrowheads indicate the type and direction of protein interconnection, respectively.

To describe their potential functions, the target genes of these two significantly and differentially deregulated EV-derived miRNAs were screened using MicroRNA Target Prediction Database (miRDB). The entire signature comprised 1781 genes, and their functional enrichment analysis was performed by EnrichR, and includes biological processes (BPs, Figure 4A), molecular functions (MFs, Figure 4B), and cellular components (CCs, Figure 4C). The top 10 BPs, MFs, and CCs are summarized in Supplementary Tables S1–S3, respectively. In particular, BPs indicated that the majority of proteins modulated by the two miRNAs were involved in the regulation of transcription, while the most abundant categories of MFs were DNA binding-related ontologies and protein serine/threonine kinase activity. CCs confirmed that most target genes act in the intracellular membrane-bound organelles, which is a GO term that includes vesicles in its GO definition. 

To obtain more stringent criteria, the MIENTURNET tool was utilized to design a network of target genes by setting the two validated miRNA-27a and miRNA-128 as the inputs. The MIENTURNET miRNA target interaction network built on the mirTarBase database identified 18 target genes of the two deregulated miRNAs. In particular, miRNA-27a interacts with *SP1*, *PLAG1*, *HOXA10*, *LDLR*, *SLC7A11*, and *YWHAZ*; whereas miRNA-128 interacted with *ADORA2B*, *PDK1*, and *BMI-1*. *ABCA1*, *WEE1*, *MAPK14*, *SMAD2*, *EGFR*, *RXRA*, *FBXW7*, and *IGF-1* were the common genes between the two miRNAs (Figure 4D). Details of the Mienturnet network are provided in Supplementary Table S4. MetaCore direct interaction network analysis of the 18 derived MIENTURNET target genes unraveled the presence of 5 central hubs, namely *SP1*, *EGFR*, *ABCA1*, *RXRA*, and *BMI-1*, which appear to be the most relevant in this ‘in silico’ analysis (Figure 4E).

**Figure 5:**
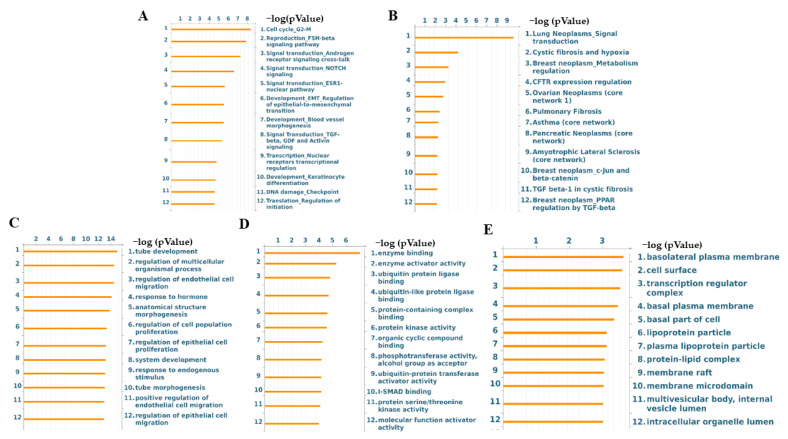
MetaCore gene enrichment analysis of 18 target genes obtained by MIENTURNET analysis. Top 12 most significantly enriched pathways for (**A**) process networks, (**B**) disease biomarker networks, (**C**) GO processes, (**D**) GO molecular functions, and (**E**) GO localization ontologies are listed.

Then, we assessed the score and priority of the network and pathways according to the relevance of the 18 target genes, prioritized by their statistical significance, using MetaCore enrichment analysis. The top 12 most significantly enriched pathways for the process networks, disease biomarker networks, GO processes, GO molecular functions, and GO localization ontologies are shown in Figure 5.

The process network category indicated that the most significantly enriched pathway was related to the cell cycle, followed by the FHS beta signaling pathway, signal transduction of androgen receptor signaling cross-talk, NOTCH signaling, ESR1 pathways, EMT transition, and blood vessel morphogenesis, all pathways closely related to breast cancer progression. MetaCore disease biomarker network analysis revealed that, among the first 12 disease annotations, six reported the involvement of target genes in different types of cancer, such as lung, breast, ovarian, and pancreatic cancer. Interestingly, GO processes showed that five of the top twelve annotations were related to cell proliferation and migration, while GO molecular functions indicated a modulation of enzyme binding and activity, together with ubiquitin ligase, binding, and transferase activities, together with protein serine/threonine kinase activity. Finally, GO localization indicated that processes are mainly modulated on the basolateral basal plasma membrane and cell surface, which are enriched by lipid rafts. Notably, among the top 12 annotations, we found multivesicular bodies, which are the intracellular compartments in which EVs are generated.

### 2.4. Clinical Value of miRNA-27a and miRNA-128 in Breast Cancer Patients

**Figure 6:**
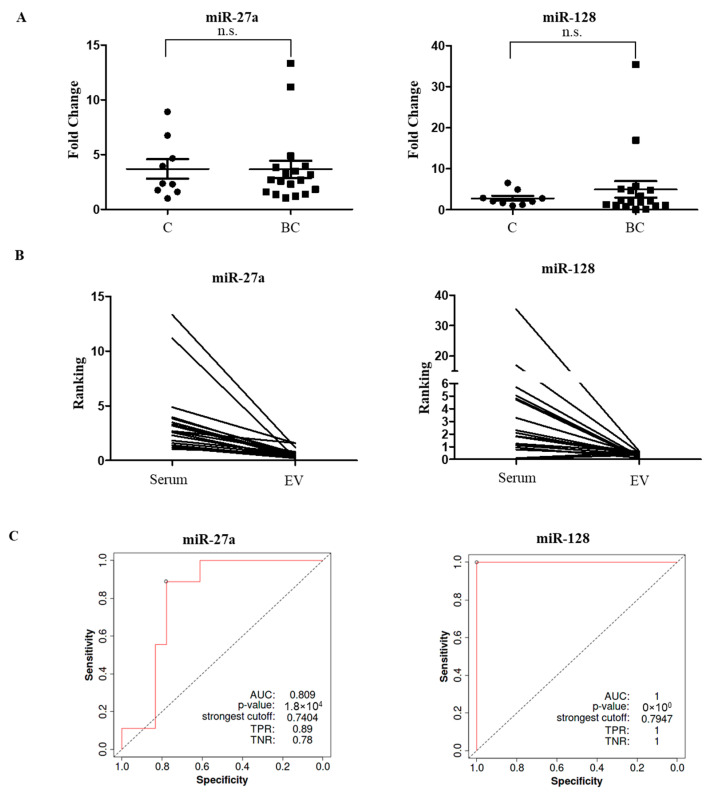
Diagnostic potential of circulating EV-derived miRNA-27a and miRNA-128 in breast cancer. (**A**). qRT-PCR was utilized to assess the levels of freely circulating miRNA-27a and miRNA-128 in the serum samples of healthy subjects (C) and breast cancer patients (BC). n.s. = not significant. (**B**). A paired dot plot shows the ranking of breast cancer patients according to miRNA-27a and miRNA-128 levels in EVs and serum; lines connect samples from the same individual. (**C**). Receiver operating characteristic (ROC) curves plotted for diagnostic potential of serum EV-derived miRNA-27a, and miRNA-128 in discriminating patients with breast cancer from healthy individuals. The corresponding AUC, sensitivity (TPR), and specificity (TNR) values are reported.

To verify whether the EV-derived miRNAs that were differentially expressed were also freely released in the serum, we analyzed miRNA-27a and miRNA-128 levels in the serum samples of the validation cohort. As shown in Figure 6A, no significant differences were found in the levels of these circulating miRNAs between the healthy subjects and patients with breast cancer. Spearman analysis revealed no correlation between EV-enclosed miRNAs and serum cell-free miRNAs (miRNA-27a: r = 0.28, *p* = 0.16; miRNA-128: r = 0.098, *p* = 0.62). The paired dot plot in Figure 6B shows the discordance in miRNA-27a and miRNA-128 levels in EVs and serum from the same patients, supporting the idea that EV-enclosed miRNA profiles may differ from cell-free miRNA profiles in the serum. 

To explore whether serum EV-derived miRNA-27a and miRNA-128 may function as potential diagnostic biomarkers in breast cancer patients, we generated ROC curves and calculated the area under the curve (AUC) using PCR data from the validation cohort (Figure 6C). ROC analyses revealed that circulating EV-derived miRNA-27a could discriminate between patients with breast cancer (n = 18) and healthy subjects (n = 9), presenting an AUC of 0.809, with a true positive rate (TPR) of 0.89, and a true negative rate (TNR) of 0.78. Interestingly, EV-miRNA-128 exhibited the best individual discriminatory power for breast cancer diagnosis (AUC = 1, TPR = 1, TNR = 1).

Finally, the association between patient clinicopathological parameters and the expression data was evaluated for the two miRNAs. There is no association between HER2 status and grade (*p* value not significant), but there is a correlation (correlation coefficient over 0.2) with estrogen receptor, progesterone receptor, and Ki-67 expression (Table 4).

These results suggest that purified circulating EV-derived miRNA-27a and miRNA-128, rather than bulk serum miRNAs, could be used to diagnose breast cancer with high accuracy.

### 2.5. Systematic Review of the Diagnostic Value of miRNA-27a and miRNA-128 in Breast Cancer Cohorts

**Figure 7:**
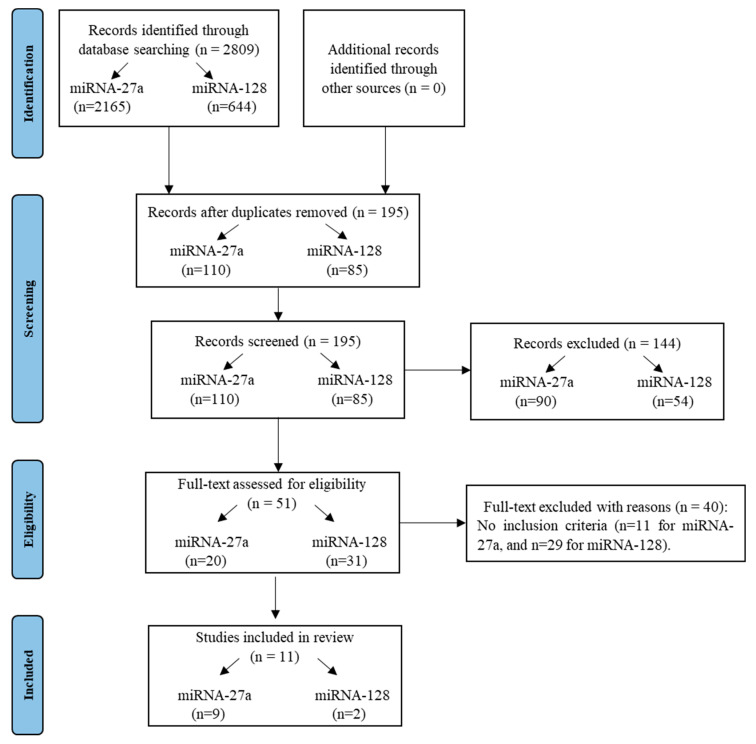
PRISMA flowchart outlining search strategy implementation and results at each stage.

Finally, a systematic review was conducted to provide an overview of the role of the two identified miRNAs in breast cancer, and to validate their possible use as non-invasive biomarkers for diagnostic purposes. Therefore, we performed a literature review to evaluate the results of all available articles concerning the expression of miRNA-27a and miRNA-128 in human breast cancers, focusing only on studies conducted in biofluid samples. After screening the titles and abstracts of all of the citations obtained by our initial literature search (Figure 7), a total of 51 full-text articles were retrieved. Eleven of them fulfilled the inclusion criteria described in the “Methods” section and were eligible for the systematic review. No studies have been found on blood EV-derived miRNA-27a and miRNA-128 in breast cancer.

All articles included in the final review are reported in Table 5, where the results of each study are summarized in terms of biofluid type and comparison of the miRNA levels in the selected population [32,33,34,35,36,37,38,39,40,41,42].

The studies included in the systematic review assessing the expression of circulating miRNA-27a in patients with breast cancer appear to be controversial. The levels of serum miRNA-27a, along with those of miRNA-451, miRNA-148a, and miRNA-30b, were found to be significantly down-regulated in breast cancer patients compared to those in benign breast tumor patients and healthy volunteers [37]. Interestingly, this panel of miRNAs was able to discriminate breast cancer patients from healthy controls, with an AUC of 95.3%, a sensitivity of 94.7%, and a specificity of 82.8%. Consistent with these results, reduced levels of miRNA-27a were also detected in 137 plasma samples from non-familial breast cancer patients [38]. Conversely, other studies have demonstrated increased expression of miRNA-27a in patients compared to controls [32,33,34,35,39,40], with a significant association with advanced clinical stage and histological grading [35,40]. Moreover, miRNA-27a relative expression levels seem to negatively correlate with hormonal receptor status [35]. 

According to our systematic review, miRNA-128 expression in biofluids appears to be underinvestigated. Indeed, only two studies have been conducted using blood samples and have reported conflicting results. Zhang et al. reported a non-significant difference in the expression of miRNA-128 in the sera of breast cancer patients and controls [42]. More recently, circulating miRNA-128 has been identified as a potential biomarker for the detection of breast cancer in high-risk benign tumors [41]. By profiling 36 plasma samples collected from patients with early-stage breast cancer and high-risk, moderate-risk, and no-risk benign breast tumors (mean age of 54.2 to 59.2 years, each group), the down-regulation of this miRNA was found in breast cancer patients, compared to that in high-risk women, with a diagnostic power above 70% (AUC > 0.7). 

Therefore, prospective studies on larger cohorts of patients are required to better verify the potential diagnostic roles of cell-free miRNA-27a and/or miRNA-128 (circulating or EV-derived) as sensitive and specific noninvasive molecular biomarkers, especially in relation to tumor subtypes.

## 3. Discussion

EV-derived miRNAs, composed of cell-free nucleic acids, have become a research hotspot as novel functional biomarkers for cancer diagnosis. Here, we performed a comprehensive analysis of circulating EV-derived miRNAs isolated from breast cancer patients and healthy subjects by taking advantage of NGS technology and verified their diagnostic accuracy. The levels of two EV miRNAs, miRNA-27a and miRNA-128, were remarkably lower in the serum of women with breast cancer than that of healthy volunteers and were not associated with circulating ones. Importantly, the ROC analyses highlighted the potential diagnostic role of these two miRNAs in breast cancer. Based on our systematic review, these findings provide the first evidence for exploiting EV miRNA-27a and miRNA-128 as promising candidates for the development of non-invasive methods for breast cancer detection.

miRNA-27a, which is transcribed by the miR-27a gene located on chromosome 19p13.13, is an important member of the miRNA family. Studies of its expression and biological functions in various tumors are diverse and inconclusive. Several reports have shown that miRNA-27a may function as an oncogene, with up-regulated levels in breast [43], colon [44,45,46], and hepatocellular [47] cancers. miRNA-27a was markedly up-regulated in invasive breast cancers that expressed low levels of its target gene *ZBTB10* (Zinc Finger and BTB Domain Containing 10), a gene involved in the regulation of transcription by RNA polymerase II, and the expression of miRNA-27a or ZBTB10 was significantly correlated with clinicopathological parameters, including tumor size, lymph node involvement, and distant metastasis, but not with receptor status [48]. Increased expression of miRNA-27a in tissues also predicts poor prognosis in patients with other tumors [48,49]. For instance, the log-rank test and Kaplan-Meier survival analyses showed that multiple myeloma patients having high miRNA-27a levels experienced a significantly shorter overall survival than those with reduced expression [49]. However, miRNA-27a down-regulation has been reported in cutaneous squamous cell carcinoma [50] as well as in colorectal cancer tissues, and these reduced levels are associated with advanced stage and distant metastasis [51]. Increased or decreased levels of miRNA-27a have also been reported in the serum of patients with breast cancer, with different correlations with clinicopathological parameters, as evidenced by our systematic review. Our results showed a significant decrease in the expression of miRNA-27a within EVs in both the breast cancer screening and validation cohorts compared to healthy controls. ROC analyses revealed that circulating EV-derived miRNA-27a could discriminate breast cancer patients from healthy subjects, with a favorable AUC value, suggesting that it might help in the diagnosis of breast cancer. Moreover, its expression did not correlate with HER2 status and grade, while it was associated with hormone receptor and ki76 levels.

miRNA-128 is an intronic miRNA produced in two major transcripts through two distinct genes, miR-128-1 and miR-128-2, able to translate into an equivalent mature miRNA sequence. This miRNA has been identified in the thymus, skeletal muscle, and brain, with high concentrations during neural development [52]. In addition to its physiological involvement in normal cellular processes, miRNA-128 plays an important regulatory role in a variety of cancers, including glioblastoma, leukemia, thyroid, lung and breast cancers [53]. The expression of miRNA-128 was found to be reduced in the majority of cancer tissues compared to that in healthy control tissues and correlated with shorter survival [54,55,56,57,58,59,60], although increased miRNA-128 levels have also been evidenced in a few reports [61,62]. Indeed, miRNA-128-3p levels in whole blood or tissues of lung cancer patients or early-stage lung cancer patients were lowly expressed as compared with those in healthy controls [55], and its expression was significantly reduced in bladder cancer tissues compared with normal adjacent ones [60]. In breast cancer tissues, decreased amounts of miRNA-128 were associated with poor clinical therapeutic efficacy and patients’ survival rates [59,63]; whereas the TCGA clinical database revealed that miRNA-128 was overexpressed in patients and was associated with a better prognosis of breast cancer [64]. Low serum miRNA-128 levels were also identified as a potential biomarker able to detect breast cancer from high-risk benign tumors in only one study [41], further indicating a lack of research on the relationship between this circulating miRNA and breast cancer. Here, we propose, for the first time, a diagnostic value for decreased EV miRNA-128 levels in a pilot sample of patients with breast cancer, with 100% specificity, and sensitivity. Certainly, further investigation from larger independent studies is needed to confirm their clinical relevance.

From a molecular point of view, it has been shown that miRNA-27a plays an important role in cell proliferation, tumor cell metabolism, immune response, and epithelial-mesenchymal transition (EMT) by regulating the activity of important oncogenic kinase signaling, including AKT, Wnt/β-catenin, and Ras/MEK/ERK pathways [65,66,67]. However, miRNA-27a has also been shown to exhibit tumor suppressor activities [68,69,70]. For instance, Li et al. reported that miRNA-27a, by targeting transmembrane protein 170B (*TMEM170B*) and inhibiting the Wnt/β-catenin pathway could suppress breast cancer proliferation, migration, and tumorigenesis [71]. miRNA profiling of parental adherent breast cancer cells and stem cell-mimicking mammospheres also identified miRNA-27a as a master negative regulator of stemness and chemoresistance, by downregulating genes crucial for the detoxification of reactive oxygen species (ROS) and autophagy [72]. On the other hand, the aberrant expression of miRNA-128 was found to inhibit breast cancer cell proliferation [64], invasiveness [58], stem cell properties [73], glucose metabolism, and mitochondrial respiration [59]. Indeed, breast cancer stem cells exhibited a marked decrease in miRNA-128 levels, and its overexpression was found to inhibit self-renewal ‘in vitro’ and tumorigenicity ‘in vivo’ by blocking the mitotic NIMA-related kinase 2 (NEK2) [73]. In addition, miRNA-128 by targeted inhibition of the insulin receptor and insulin receptor substrate 1 negatively modulated the glucose metabolism, mitochondrial respiration, and proliferation of TNBC cells [59]. Exosomal miRNA-27a [74] and miRNA-128 [75] have also been found to regulate EMT, a crucial hallmark of cancer progression [76]. Our enrichment pathway analysis based on the target gene prediction of miRNA-27a and miRNA-128 confirmed that most target genes are related to the intercellular membrane-bound organelles, which includes vesicles in its GO definition. In addition, the majority of proteins in biological processes and molecular functions are involved in the regulation of transcription, and another abundant category of molecular function is protein serine/threonine kinase activity. Of note, various types of addiction to specific transcriptional programs and/or kinase activities appear to operate in specific subsets of cancer and are thought to deeply contribute to cancer pathogenesis [77,78]. Interestingly, when a more accurate and stringent analysis was performed on the regulated target genes of these two candidate miRNAs, Mienturnet analysis followed by MetaCore process networks revealed the presence of important central hubs, including the transcriptional factors SP1 (specificity protein 1), RXRA (retinoid X receptor alpha), and BMI-1 (polycomb ring finger proto-oncogene) and the epidermal growth factor receptor kinase EGFR, with multiple roles in breast cancer development and progression [79,80,81,82]. Moreover, Metacore gene enrichment analysis indicated the involvement of the 18 target genes in cell cycle transition from G2 to M, the follicle-stimulating hormone (FSH) beta signaling pathway, androgen receptor signaling cross-talk, NOTCH signaling, ESR1 nuclear pathway and signaling, and EMT among the top enriched processes. A high G2M pathway score is associated with aggressive clinical features, survival, and treatment response in breast cancer [83]. FSH binds to its own receptor, and interacts with breast cancer cells, promoting their motility, invasion [84], and chemoresistance [85]. Activation of the androgen receptor, NOTCH, and ESR1 signaling affects several aspects of breast carcinogenesis, from tumor development to progression and metastatic outgrowth through genomic and non-genomic pathways [86,87,88]. At present, no experimental evidence indicates a direct correlation between the two EV-deregulated miRNAs and the previously mentioned MetaCore ontologies, suggesting that this could be an active area of investigation that may deserve our future attention. Indeed, an improved understanding of the mechanisms that create such transcription and/or kinase dependencies (i.e., miRNA regulatory networks) in the context of cancer cell heterogeneity may be important for the development of effective therapies for personalized medicine.

Currently, it is still debated whether EV-derived miRNA detection assays are superior to whole serum-based assays, as the EV membrane may protect the RNA cargo from degradation in the bloodstream and the intraluminal RNA content becomes relatively more stable [12]. Here, we performed a systematic comparison of miRNA levels in the serum and EVs isolated from the same samples in a well-characterized cohort of breast cancer patients and healthy subjects. Our results showed that serum miRNA-27a and miRNA-128 levels did not differ between patients and controls. Interestingly, the EV-incorporated and serum miRNA profiles were clearly different, suggesting that purified serum-EV miRNA-27a and miRNA-128, rather than bulk serum miRNAs, could be used to diagnose breast cancer with high accuracy. This is in line with other studies indicating a poor correlation between miRNA levels in EVs and the blood [89,90,91]. For instance, Cheng et al. demonstrated that miRNA levels differ remarkably between plasma and serum EVs, and between EVs and cell-free plasma and serum [89]. 

In conclusion, our findings provide the first line of evidence of the role of EV-derived miRNA-27a and miRNA-128 as diagnostic markers that can discriminate with high sensitivity and specificity patients with breast cancer from healthy subjects. Of note, the miRNA profiling of EVs purified from liquid biopsies of breast cancer using small RNA-sequencing as a screening method, followed by RT-qPCR analysis of selected miRNAs can be considered as a valid approach to identify the miRNAs involved in cancer pathways. The results from these assays can be used, after further validation in a larger cohort, to augment currently available breast cancer diagnostic and prognostic methods. Nevertheless, challenges remain before the levels of these two miRNAs within EVs can be leveraged as bona fide biomarkers in breast cancer. First, bioinformatics-predicted pathways need experimental verification to better clarify the role of these EV-derived miRNAs in breast carcinogenesis. Secondly, the inconsistent expression of EV-miRNA-27a and miRNA-128 versus circulating miRNAs deserves a deeper evaluation. More importantly, the relatively limited sample size of our population cohort within a single hospital may hamper definitive conclusions, and further investigation from larger independent studies will help to unveil their clinical value, improving the statistical power of the results. Certainly, to facilitate the bench to bedside transition, the technical sources of variation among studies, including the choice of serum or plasma samples, EV isolation techniques, RNA extraction methods, miRNA normalization (i.e., reliable endogenous genes for normalization of EV miRNAs), technological platform (i.e., microarray, qRT-PCR, NGS) and data analysis, should be minimized. 

## 4. Materials and Methods

### 4.1. Study Population

A total of 45 female patients, diagnosed with primary breast cancer at the Oncology Unit, Department of Clinical Medicine and Surgery of the University of Naples Federico II, Naples, Italy, were enrolled between 2016 and 2020. All enrolled breast cancer patients had non-metastatic disease and were eligible for neoadjuvant systemic treatment. A cohort of 36 blood female donors with no history of malignant disease was recruited from the Health Center, Rende, CS, Italy. In particular, the number of the subjects and their clinicopathological characteristics are summarized in Table 1 for the screening cohort (n = 27 for breast cancer patients, and n = 27 for healthy subjects), and in Table 3 for the validation cohort (n = 18 for breast cancer patients, and n = 9 for healthy subjects).

The study protocol was performed in accordance with the ethical guidelines of the Declaration of Helsinki and was approved by the Ethical Committee of the University of Naples Federico II (protocol number: 107/05) and the University of Calabria (protocol number: 4086/19). Written informed consent was obtained from each enrolled subject. Data for the following clinical variables were recorded: age, histology, grading, estrogen receptor (ER), progesterone receptor (PgR), human epidermal growth factor receptor 2 (HER2) status, and Ki-67 levels. 

### 4.2. Blood Sample Collection and Extracellular Vesicles Isolation

Peripheral blood samples were collected after 8–10 h of overnight fasting conditions. Blood samples were centrifuged at 3000× rpm for 10 min, and the obtained serum fractions were stored in sterile tubes at −80 °C until further processing. EVs were isolated from serum samples (250 µL) using the polymer-based precipitation method (ExoQuick, System Bioscience, Palo Alto, CA, USA), according to the manufacturer’s instructions, as previously described [92]. The pellet obtained was resuspended in sterile phosphate-buffered saline (PBS) or RIPA lysis buffer, for immunoblot protein control.

The EV samples of the screening cohort have been mixed in equal amounts (3 samples in each pool) to obtain pooled EVs for breast cancer patients (n = 9 pools), and healthy controls (n = 9 pools). The pooled samples allowed for obtaining enough RNA to yield reproducible reads for miRNA profiling. The samples were stored at −80 °C until further analysis. A schematic representation of this process is reported in Figure 1A.

### 4.3. Transmission Electron Microscopy (TEM) 

Serum-derived EVs were suspended in PBS, fixed in 3% glutaraldehyde, and transferred to formovar-coated grids for 20 min. Subsequently, they were treated with 2% uranyl for 1 min, dried, and examined under a Jeol JEM 1400 Plus electron microscope (Jeol Ltd., Milano, Italy) at 80 kV. 

### 4.4. Nanoparticle Tracking Analysis (NTA) 

The size distribution and particle concentration (particles/mL) of EV samples were analyzed using a NanoSight NS300 by Alfatest srl (Cernusco sul Naviglio, Milan, Italy) instrument and Nanosight particle tracking software (NS300, Malvern Panalytical Ltd., Malvern, UK), as previously described [93]. 

### 4.5. Immunoblot Analysis

EVs were lysed in RIPA buffer, as described previously [94]. The BCA Protein Assay Kit (Thermo Fisher Scientific, Waltham, MA, USA) was utilized to assess protein concentration, according to the manufacturer’s instructions. Equal amounts of protein extracts were resolved on 10% SDS–polyacrylamide and probed with anti-Tsg101 (sc-7964, Santa Cruz Biotechnology, Dallas, TX, USA), anti-CD9 (ab236630, Abcam, Cambridge, UK), anti-CD81 (ab155760, Abcam), and anti-calnexin (sc-11397, Santa Cruz Biotechnology) overnight at 4 °C, followed by secondary IRDye 800CW goat anti-mouse or anti-rabbit antibodies (LI-COR, Bad Homburg vor der Höhe, Germany). Immunoblot images were acquired using Odissey FC (LI-COR, Lincoln, NE, USA). Uncropped blots are shown in Supplementary Figure S1.

### 4.6. RNA Isolation

Total RNA was isolated from EVs and serum samples using the miRNeasy Mini Kit (Qiagen, Hilden, Germany), following the manufacturer’s instructions. Purified RNA was eluted RNase-free water and stored at −80 °C. RNA was quantified using a Qubit Fluorometer (Thermo Fisher Scientific) and its quality was assessed using the TapeStation 4200 (Agilent Technologies, Santa Clara, CA, USA). 

### 4.7. miRNA Sequencing and Data Analysis

Small RNA-seq was carried out to profile miRNAs in EVs from healthy subjects and patients with breast cancer at Genomix4Life Srl (Baronissi, SA, Italy). miRNAseq was performed to profile miRNAs in EVs from healthy subjects and patients with breast cancer. A total of 9 biological replicates for each condition were analyzed. Small RNA libraries were constructed using NEXTFLEX Small RNA-Seq Kit v3 (PerkinElmer, Waltham, MA, USA) according to the manufacturer’s instructions. Libraries were quantified, processed, and sequenced using an Illumina NextSeq 550 Dx System (Illumina, San Diego, CA, USA), as previously described [92]. The raw sequencing files were deposited in the NCBI’s Gene Expression Omnibus and are available through GEO Series accession numbers GSE239341 and GSE222681. miRNA profiling was performed as previously reported [92]. Briefly, the bioinformatics tool cutadapt (version 2.5) was used to remove the adapter sequence according to the kit used. The tool sRNAbench was used to remove low-quality reads and to obtain miRNA expression profiling with respect to the miRBase database (version22—GRCh38-GCA_000001405.15). The sequencing depth was ~30 million reads per sample. A minimal pre-filtering to keep only rows that had at least 10 reads in total was performed, as suggested by the authors of the Deseq2-Bioconductor package. To identify differentially expressed miRNAs, the DESeq2 algorithm was used and miRNAs with padj ≤ 0.05, and |fold-change| (|FC|) ≥ 1.5 were considered to be differentially expressed between the two groups.

### 4.8. qRT-PCR Analysis

qRT-PCR was carried out in RNA samples isolated from circulating EVs and serum samples from the validation cohort of healthy subjects and patients with breast cancer. The TaqMan™ Advanced miRNA cDNA Synthesis Kit (Applied Biosystems, Thermo Fisher Scientific) was used to obtain cDNA. miRNA expression levels were quantified using TaqMan™ MicroRNA assay probes and TaqMan™ Universal PCR Master Mix (Applied Biosystems) according to the manufacturer’s instructions, as previously described [92]. The adopted threshold value was 0.2. The spike-in cel-miR-39, the endogenous miRNA-191, or miRNA-484 were used to normalize the data (ID name: cel-miR-39-3p & Assay name: 478293_mir, ID name: hsa-miR-484 & Assay name: 478308_mir, ID name: hsa-miR-191-5p & Assay name: 477952_mir, Thermo Fisher Scientific), as suggested by the manufacturer and other reports [92,95,96,97,98]. Relative miRNA expression levels were calculated using the comparative cycle threshold 2^−ΔΔCt^ method, using DataAssist v3.01 Software (Applied Biosystems). 

### 4.9. Identification of miRNA Target Genes and Their Molecular Pathways

The miRNA target genes were identified for selective deregulated miRNAs using the mirDB online database [99] with default parameters. Then, the list of target genes was processed using EnrichR to perform pathway enrichment analyses for biological processes, molecular functions, and cellular components. The top ten GO terms were considered. 

### 4.10. Construction of the Regulatory Network

MicroRNA ENrichment TURned NETwork (MIENTURNET, Rome, Italy) was used to build and analyze the miRNA-target interaction network [100]. In the current study, we uploaded deregulated miRNAs obtained by RNA-Seq and validated by qRT-PCR to the MIENTURNET web tool (http://userver.bio.uniroma1.it/apps/mienturnet/ accessed on 8 May 2023) to dissect potential miRNA activity. Networks for miRNA interactions and targets were produced using the miRTarBase database. To obtain more stringent criteria, we set the following MIENTURNET parameters: threshold for the minimum number of miRNA-target interactions = 2, and threshold for the adjusted *p*-value (FDR) = 1. We also set filter by evidence category ‘Strong’. 

### 4.11. MetaCore Functional Analysis

The regulatory network was processed using MetaCore™ software for functional data analysis (https://portal.genego.com/, accessed on 15 May 2023, Clarivate Analytics, London, UK). The gene name list obtained by MIENTURENET was imported into MetaCore and processed for functional enrichment and the construction of networks of corresponding proteins that directly interact with each other, according to the manually curated MetaCore in-house database of physiological and pathological protein–protein and protein–nucleic acid interactions, signaling, and metabolic pathways. Pathway enrichment analysis using MetaCore Enrichment Analysis tool allowed us to match the input genes in MetaCore functional ontologies. A random interaction between a gene list and an ontology was then estimated by the *p*-value, with a lower *p*-value corresponding to the higher relevance of the determined pathway. The direct interaction networks (DIN) of the analysis represented the interactomic core of the investigated process and were ordered by their statistical significance. Nets were then graphically visualized as nodes (proteins) and edges (interconnections among proteins). Functional enrichment was built using process network and disease by biomarker ontologies and by GO processes, GO molecular functions and GO localizations public ontologies.

### 4.12. Systematic Review Design

The present study followed the Preferred Reporting Items for Systematic Review and Meta-Analysis (PRISMA) [101]. An online literature search for miRNA-128 and miRNA-27a was conducted in the PubMed database between 7 May 2023, and 5 June 2023 to obtain all studies related to the predictive diagnostic performance of these miRNAs in human breast cancers. Our search strategy was based on a combination of keywords and Boolean operators organized as it follows: (1) MicroRNA* OR “Micro RNA” OR “RNA Micro” OR miRNA* OR pri-miRNA* OR stRNA* OR pre-miRNA*; (2) Small AND Temporal AND RNA*; (3) 1 OR 2; (4) 128; (5) 3 AND 4; (6) miR-128 OR microRNA-128 OR hsa-mir-128 OR miRNA-128 OR micro-RNA-128 OR pri-miRNA-128 OR stRNA-128 OR pre-miRNA-128; (7) 5 OR 6; (8) “MicroRNAs” [Mesh]) AND 128; (9) 7 OR 8; (10) neoplas* OR tumo* OR cancer*; (11) 9 AND 10. The same strategy was used to replicate miRNA-27a expression. Two review authors (I.B. and S.C.) independently screened the titles and abstracts to identify all available articles for eligibility. A publication filter was not applied to the database. Following this, the review authors independently performed a full-text review of the articles identified through screening. Screening for titles and abstracts and full-text screening were performed using a standardized form with explicitly predefined inclusion and exclusion criteria. A manual search of study references was performed for completeness. Studies were considered for inclusion if they met the following criteria: (1) full original articles published in English; (2) articles concerning the evaluation of cell-free miRNAs in human biofluids from breast cancer patients; (3) recruitment of more than 10 participants for the study; (4) sample collection before surgical resection or other therapeutic treatments; (5) no declared concomitance with other diseases; and (6) analysis of miRNA levels for diagnostic purposes. Exclusion criteria were: (1) studies showing duplicate databases; (2) reviews of the literature, editorial notes, posters, clinical case reports, meta-analyses, conference abstracts, letters to the editor, theses, dissertations, and book chapters; (3) and experiments without a control group or comparison group. Any disagreements between the reviewers were resolved through discussions and mutual consent between reviewers.

### 4.13. Statistical Analysis

Receiver operating curves (ROC) were constructed and the area under the curve (AUC) with a 95% confidence interval, sensitivity, and specificity were calculated to evaluate the diagnostic accuracy of miRNAs. Spearman correlation analysis was used to determine the correlation between EV-enclosed miRNAs and serum cell-free miRNAs in patients with breast cancer. The paired dot plot was built using the results from qRT-PCR analysis in the validation cohort analyzed by GraphPad-Prism 7 software. Association between EV-miRNA-27a and EV-miRNA-128 and the clinicopathological data of patients with breast cancer were performed using Mann–Whitney and Kruskal–Wallis tests for categorical variables and with Pearson correlation analysis for continuous variables. Data were analyzed for statistical significance (*p* < 0.05) using a two-tailed Student’s test, performed using GraphPad-Prism ver. 7 (GraphPad Software Inc., San Diego, CA, USA).

## Figures and Tables

**Table 1:** Clinical characteristics of breast cancer patients and healthy controls.

Clinical Variables	Breast Cancer Patients (BC)	Healthy Subjects (C)
**Sample Size (n)**	**27**	**27**
*Age*, *y*		
Median	50	51
Range	30–76	23–61
*Histology*, *%*		
In situ ductal carcinoma	3.7	
Invasive ductal carcinoma	81.5	
Invasive lobular carcinoma	7.4	
*Grading*, *%*		
Well differentiated (G1)	7.4	
Moderately differentiated (G2)	33.3	
Poor/undifferentiated (G3)	33.3	
Unknown	25.9	
*Estrogen receptor status*, *%*		
Negative	11	
Positive (>1%)	89	
*Progesterone receptor status*, *%*		
Negative	18.5	
Positive (>1%)	78	
*HER2/Neu status*, *%*		
Negative	55.5	
Positive	41	
*Ki-67*, *%*		
<14	7.4	
≥14	89	

y, years; HER2/Neu, Human Epidermal Growth Factor 2.

**Table 2:** List of differentially expressed mature miRNAs in EVs derived from breast cancer patients compared to healthy controls.

miRNAs	Fold Change	padj
hsa-miR-28-5p	102.18	2.59 × 10^7^
hsa-miR-27a-3p	−114.83	4.60 × 10^7^
hsa-miR-128-3p	−112.14	2.30 × 10^5^
hsa-miR-15b-5p	−1.88	1.00 × 10^4^
hsa-miR-181a-5p	−1.90	1.28 × 10^3^
hsa-miR-148a-3p	−1.74	1.29 × 10^3^

**Table 3:** Clinical characteristics of breast cancer patients and healthy controls of the validation cohort.

Clinical Variables	Breast Cancer Patients (BC)	Healthy Controls (C)
**Sample Size (n)**	**18**	**9**
*Age*, *y*		
Median	57	41
Range	33–79	26–54
*Histology*, *%*		
In Situ ductal carcinoma	-	
Invasive ductal carcinoma	83.3	
Invasive lobular carcinoma	5	
*Grading*, *%*		
Well differentiated (G1)	5.5	
Moderately differentiated (G2)	22.2	
Poor/undifferentiated (G3)	50	
Unknown	11.1	
*Estrogen receptor status*, *%*		
Negative	22.2	
Positive (>1%)	77.8	
*Progesterone receptor status*, *%*		
Negative	33.3	
Positive (>1%)	66.7	
*HER2/Neu status*, *%*		
Negative	88.8	
Positive	11.1	
*Ki-67*, *%*		
<14	11.1	
≥14	83.3	

y, years; HER2/Neu, Human Epidermal Growth Factor 2.

**Table 4:** Relationship of EV-miRNA-27a and EV-miRNA-128 and clinicopathological data of breast cancer patients.

	miRNA-27a	miRNA-128	Combined
HER2 status (Mann-Whitney, *p*-value)	0.21	0.1	0.17
Grade (Kruskal-Wallis, *p*-value)	0.22	0.4	0.42
Estrogen Receptor Expression (Pearson correlation coefficient)	0.39	0.23	0.25
Progesterone Receptor Expression (Pearson correlation coefficient)	0.39	0.30	0.44
Ki-67 Expression (Pearson correlation coefficient)	0.33	0.40	0.38

HER2/neu, Human Epidermal Growth Factor 2.

**Table 5:** Studies included in the systematic review.

MicroRNA	Biofluid Type	Deregulation	Reference
**miRNA-27a**	Serum	↑ BC vs. C	[32]
	Plasma	↑ BC vs. C	[33]
		↑ Late BC vs. early BC	
		=Benign vs. C vs. high risk BC	
		=high risk BC vs. C	
	Plasma	↑ BC vs. C	[34]
	Serum	↑ Primary BC vs. benign breast lesions	[35]
		↑ Primary BC vs. C	
		↑ Benign breast lesions vs. C	
	Plasma	↑ BC vs. C	[36]
		↓ BC after chemotherapy vs. BC before chemotherapy	
		↑ BC after chemotherapy vs. C	
	Serum	↓ BC vs. C	[37]
	Plasma	↓ BC vs. C	[38]
	Serum	↑ BC vs. C	[39]
	Plasma	↑ BC vs. C	[40]
**miRNA-128**	Plasma	↓ BC vs. high-risk BC	[41]
	Serum	=BC vs. C	[42]

BC, Breast Cancer. C, healthy controls.

## Data Availability

The data presented in this study are openly available in the NCBI’s Gene Expression Omnibus and are available through GEO Series accession numbers GSE239341 and GSE222681.

## Supplementary Materials

The following supporting information can be downloaded at: https://www.mdpi.com/article/10.3390/ijms242115695/s1.
