# Supplementary material for: miRNAs in the Box: Potential Diagnostic Role for Extracellular Vesicle-Packaged miRNA-27a and miRNA-128 in Breast Cancer
Source: Int J Mol Sci. 2023 Oct 28;24(21):15695. doi: 10.3390/ijms242115695 (PMC10649351; doi:10.3390/ijms242115695)
Supplement: Supplementary file 1 [file ijms-24-15695-s001.zip › ijms-2614594-supplementary/Suppl Tables and Figure/Supplemenatry Table S1.docx]

**Supplementary Table 1. Top 10 Biological Processes identified in functional enrichment analysis performed by EnrichR.**

| **Term** | **Overlap** | **p-value** | **Adjusted p-value** | **Odds Ratio** | **Combined Score** |
| --- | --- | --- | --- | --- | --- |
| *Regulation Of DNA-templated Transcription (GO:0006355)* | 310/1922 | 4.33E-27 | 1.77E-23 | 2.17 | 131.79 |
| *Regulation Of Transcription By RNA Polymerase II (GO:0006357)* | 309/2028 | 8.19E-23 | 1.67E-19 | 2.01 | 102.47 |
| *Positive Regulation Of DNA-templated Transcription (GO:0045893)* | 208/1243 | 6.30E-20 | 8.57E-17 | 2.19 | 97.06 |
| *Positive Regulation Of Transcription By RNA Polymerase II (GO:0045944)* | 165/938 | 5.11E-18 | 5.21E-15 | 2.30 | 91.75 |
| *Regulation Of Gene Expression (GO:0010468)* | 177/1127 | 2.40E-14 | 1.96E-11 | 2.00 | 62.91 |
| *Positive Regulation Of Nucleic Acid-Templated Transcription (GO:1903508)* | 105/557 | 8.69E-14 | 5.91E-11 | 2.46 | 74.05 |
| *Negative Regulation Of DNA-templated Transcription (GO:0045892)* | 162/1025 | 1.94E-13 | 1.13E-10 | 2.01 | 58.90 |
| *Peptidyl-Serine Modification (GO:0018209)* | 44/166 | 2.49E-11 | 1.27E-08 | 3.75 | 91.74 |
| *Protein Phosphorylation (GO:0006468)* | 91/500 | 3.01E-11 | 1.36E-08 | 2.34 | 56.80 |
| *Protein Modification Process (GO:0036211)* | 117/711 | 4.09E-11 | 1.67E-08 | 2.08 | 49.90 |

p values from Fisher’s exact test; adjusted p-values computed using Benjamini-Hochberg method for correction for multiple hypotheses testing.
