# Supplementary material for: miRNAs in the Box: Potential Diagnostic Role for Extracellular Vesicle-Packaged miRNA-27a and miRNA-128 in Breast Cancer
Source: Int J Mol Sci. 2023 Oct 28;24(21):15695. doi: 10.3390/ijms242115695 (PMC10649351; doi:10.3390/ijms242115695)
Supplement: Supplementary file 1 [file ijms-24-15695-s001.zip › ijms-2614594-supplementary/Suppl Tables and Figure/Supplementary Table S2.docx]

**Supplementary Table 2. Top 10 Molecular Function identified in functional enrichment analysis performed by EnrichR.**

| **Term** | **Overlap** | **p-value** | **Adjusted p-value** | **Odds Ratio** | **Combined Score** |
| --- | --- | --- | --- | --- | --- |
| *Sequence-Specific DNA Binding (GO:0043565)* | 121/717 | 3.37E-12 | 2.73E-09 | 2.15 | 56.93 |
| *Protein Serine/Threonine Kinase Activity (GO:0004674)* | 69/342 | 7.66E-11 | 3.10E-08 | 2.64 | 61.71 |
| *Sequence-Specific Double-Stranded DNA Binding (GO:1990837)* | 116/715 | 1.22E-10 | 3.30E-08 | 2.04 | 46.77 |
| *Double-Stranded DNA Binding (GO:0003690)* | 102/650 | 1.01E-08 | 2.05E-06 | 1.95 | 36.06 |
| *RNA Polymerase II Transcription Regulatory Region Sequence-Specific DNA Binding (GO:0000977)* | 162/1225 | 1.43E-07 | 2.31E-05 | 1.61 | 25.45 |
| *Transcription Cis-Regulatory Region Binding (GO:0000976)* | 76/474 | 3.27E-07 | 4.41E-05 | 1.99 | 29.80 |
| *Cis-Regulatory Region Sequence-Specific DNA Binding (GO:0000987)* | 146/1098 | 4.45E-07 | 5.14E-05 | 1.61 | 23.68 |
| *RNA Polymerase II Cis-Regulatory Region Sequence-Specific DNA Binding (GO:0000978)* | 147/1122 | 9.65E-07 | 9.76E-05 | 1.59 | 22.03 |
| *DNA Binding (GO:0003677)* | 115/846 | 2.71E-06 | 2.44E-04 | 1.65 | 21.16 |
| *DNA-binding Transcription Activator Activity, RNA Polymerase II-specific (GO:0001228)* | 56/348 | 1.01E-05 | 8.16E-04 | 1.99 | 22.92 |

p values from Fisher’s exact test; adjusted p-values computed using Benjamini-Hochberg method for correction for multiple hypotheses testing.
