# Supplementary material for: miRNAs in the Box: Potential Diagnostic Role for Extracellular Vesicle-Packaged miRNA-27a and miRNA-128 in Breast Cancer
Source: Int J Mol Sci. 2023 Oct 28;24(21):15695. doi: 10.3390/ijms242115695 (PMC10649351; doi:10.3390/ijms242115695)
Supplement: Supplementary file 1 [file ijms-24-15695-s001.zip › ijms-2614594-supplementary/Suppl Tables and Figure/Supplementary Table S4.docx]

**Supplementary Table 4. Mienturnet Network Properties.**

| **Node** | **Degree** | **Closeness** | **Betweenness** | **Eccentricity** | **Clustering Coefficient** | **Average Shortest Path Lenght** |
| --- | --- | --- | --- | --- | --- | --- |
| **hsa-miR-27a-3p** | 14 | 0.679 | 0.626 | 3 | 0 | 1.40 |
| **hsa-miR-128-3p** | 12 | 0.594 | 0.468 | 3 | 0 | 1.60 |
| **EGFR** | 2 | 0.528 | 0.0256 | 2 | 0 | 1.80 |
| **FBXW7** | 2 | 0.528 | 0.0256 | 2 | 0 | 1.80 |
| **WEE1** | 2 | 0.528 | 0.0256 | 2 | 0 | 1.80 |
| **IGF1** | 2 | 0.528 | 0.0256 | 2 | 0 | 1.80 |
| **ABCA1** | 2 | 0.528 | 0.0256 | 2 | 0 | 1.80 |
| **RXRA** | 2 | 0.528 | 0.0256 | 2 | 0 | 1.80 |
| **MAPK14** | 2 | 0.528 | 0.0256 | 2 | 0 | 1.80 |
| **SMAD2** | 2 | 0.528 | 0.0256 | 2 | 0 | 1.80 |
| **SP1** | 1 | 0.413 | 0.00 | 4 | 0 | 2.30 |
| **BMI1** | 1 | 0.380 | 0.00 | 4 | 0 | 2.50 |
| **TGFBR1** | 1 | 0.380 | 0.00 | 4 | 0 | 2.50 |
| **PLAG1** | 1 | 0.413 | 0.00 | 4 | 0 | 2.30 |
| **ADORA2B** | 1 | 0.380 | 0.00 | 4 | 0 | 2.50 |
| **YWHAZ** | 1 | 0.413 | 0.00 | 4 | 0 | 2.30 |
| **SLC7A11** | 1 | 0.413 | 0.00 | 4 | 0 | 2.30 |
| **HOXA10** | 1 | 0.413 | 0.00 | 4 | 0 | 2.30 |
| **LDLR** | 1 | 0.413 | 0.00 | 4 | 0 | 2.30 |
| **PDK1** | 1 | 0.380 | 0.00 | 4 | 0 | 2.50 |

Degree: the number of incoming and outgoing edges of each node; closeness: the inverse of the average length of the shortest paths between a node and all other nodes in the network; betweenness: the number of shortest paths going through the node; eccentricity: the shortest path distance of a node from the farthest other node in the network; clustering coefficient: measurement the probability that the adjacent nodes of a node are connected; average shortest path length: the average length of the shortest paths between a node and all other nodes in the network.
