# Supplementary material for: miRNAs in the Box: Potential Diagnostic Role for Extracellular Vesicle-Packaged miRNA-27a and miRNA-128 in Breast Cancer
Source: Int J Mol Sci. 2023 Oct 28;24(21):15695. doi: 10.3390/ijms242115695 (PMC10649351; doi:10.3390/ijms242115695)
Supplement: Supplementary file 1 [file ijms-24-15695-s001.zip › ijms-2614594-supplementary/Suppl Tables and Figure/uncropped IB.pptx]

## Slide 1
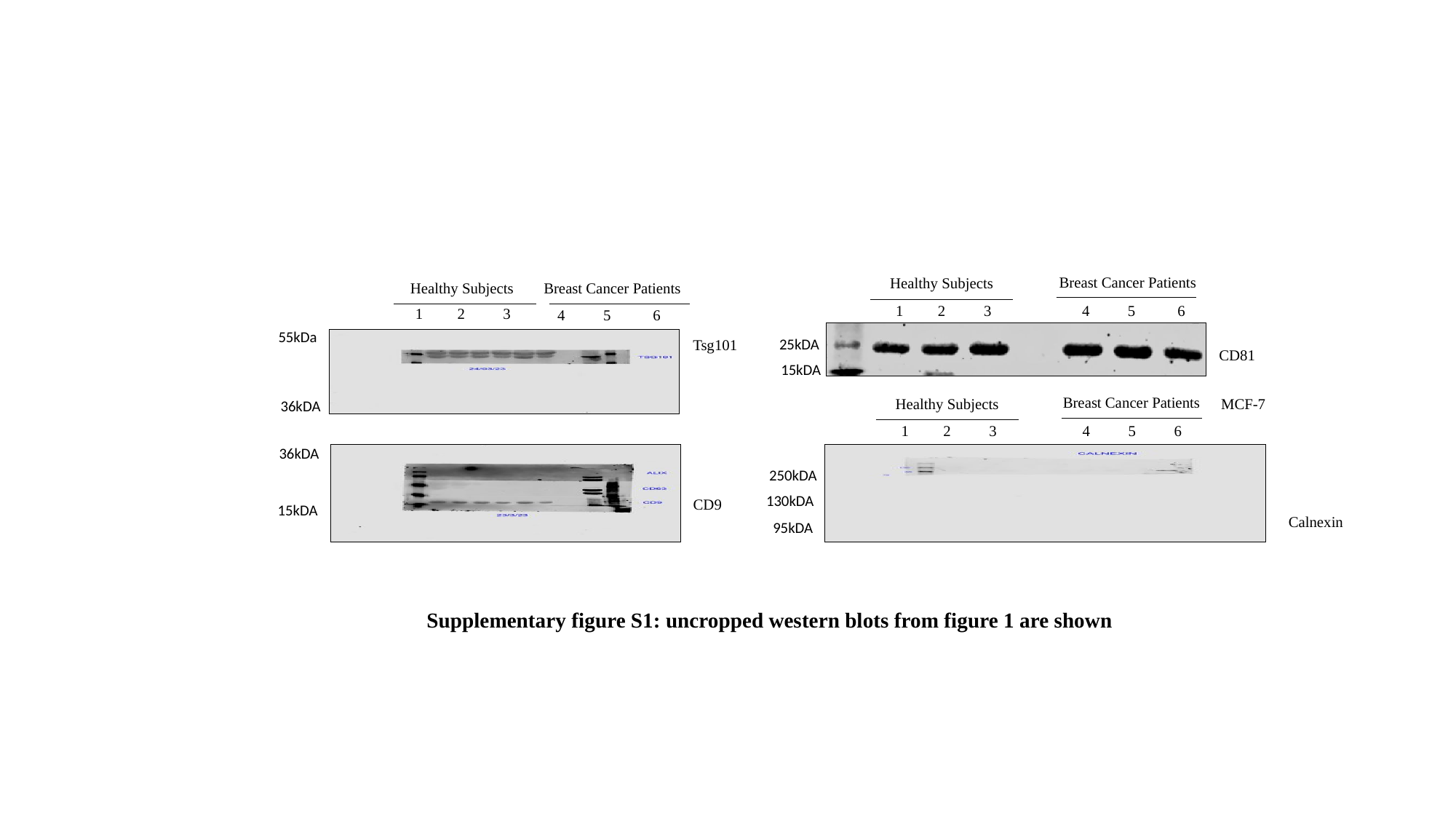

Breast Cancer Patients
Healthy Subjects
1 2 3
 4 5 6
Breast Cancer Patients
Healthy Subjects
1 2 3
4 5 6
55kDa
25kDA
Tsg101
CD81
15kDA
Breast Cancer Patients
Healthy Subjects
1 2 3
4 5 6
MCF-7
36kDA
36kDA
250kDA
130kDA
CD9
15kDA
Calnexin
95kDA
Supplementary figure S1: uncropped western blots from figure 1 are shown
