# Supplementary material for: miRNAs in the Box: Potential Diagnostic Role for Extracellular Vesicle-Packaged miRNA-27a and miRNA-128 in Breast Cancer
Source: Int J Mol Sci. 2023 Oct 28;24(21):15695. doi: 10.3390/ijms242115695 (PMC10649351; doi:10.3390/ijms242115695)
Supplement: Supplementary file 1 [file ijms-24-15695-s001.zip › ijms-2614594-supplementary/Suppl Tables and Figure/Supplementary Table S3.doc]

**Supplementary Table 3. Top 10 Cellular Component identified in functional enrichment analysis performed by EnrichR.**

| **Term** | **Overlap** | **p-value** | **Adjusted p-value** | **Odds Ratio** | **Combined Score** |
| --- | --- | --- | --- | --- | --- |
| *Intracellular Membrane-Bounded Organelle (GO:0043231)* | 609/5175 | 1.79E-16 | 5.98E-14 | 1.55 | 56.33 |
| *Nucleus (GO:0005634)* | 538/4487 | 8.91E-16 | 1.49E-13 | 1.56 | 54.20 |
| *Neuron Projection (GO:0043005)* | 84/557 | 1.11E-06 | 1.24E-04 | 1.85 | 25.46 |
| *Serine/Threonine Protein Kinase Complex (GO:1902554)* | 17/60 | 1.22E-05 | 9.32E-04 | 4.07 | 46.09 |
| *Dendrite (GO:0030425)* | 46/270 | 1.40E-05 | 9.32E-04 | 2.12 | 23.81 |
| *Axon (GO:0030424)* | 36/205 | 6.05E-05 | 0.003366 | 2.20 | 21.40 |
| *Activin Receptor Complex (GO:0048179)* | 04/05 | 2.91E-04 | 0.013204 | 41.00 | 333.87 |
| *Postsynaptic Specialization Membrane (GO:0099634)* | 11/38 | 3.39E-04 | 0.013204 | 4.18 | 33.45 |
| *Postsynaptic Density (GO:0014069)* | 27/151 | 3.56E-04 | 0.013204 | 2.24 | 17.83 |
| *Postsynaptic Density Membrane (GO:0098839)* | 10/34 | 5.45E-04 | 0.018216 | 4.28 | 32.16 |

p values from Fisher’s exact test; adjusted p-values computed using Benjamini-Hochberg method for correction for multiple hypotheses testing.
